# Identification and analysis of lignin biosynthesis genes related to fruit ripening and stress response in banana (*Musa acuminata* L. AAA group, cv. Cavendish)

**DOI:** 10.3389/fpls.2023.1072086

**Published:** 2023-03-22

**Authors:** Zhuo Wang, Xiao-ming Yao, Cai-hong Jia, Bi-yu Xu, Jing-yi Wang, Ju-hua Liu, Zhi-qiang Jin

**Affiliations:** ^1^ Key Laboratory of Tropical Crop Biotechnology of Ministry of Agriculture and Rural Affairs, Institute of Tropical Bioscience and Biotechnology, Chinese Academy of Tropical Agricultural Sciences, Haikou, Hainan, China; ^2^ Hainan Academy of Tropical Agricultural Resource, Chinese Academy of Tropical Agricultural Sciences, Haikou, Hainan, China; ^3^ Sanya Research Institute of Chinese Academy of Tropical Agricultural Sciences, Sanya, Hainan, China; ^4^ Beijing Genomics Institute (BGI)-Sanya, Beijing Genomics Institute (BGI)-Shenzhen, Sanya, China

**Keywords:** lignin biosynthesis genes, banana, fruit ripening, stresses, co-expression networks, expression analysis

## Abstract

**Background:**

Lignin is a key component of the secondary cell wall of plants, providing mechanical support and facilitating water transport as well as having important impact effects in response to a variety of biological and abiotic stresses.

**Results:**

In this study, we identified 104 genes from ten enzyme gene families related to lignin biosynthesis in *Musa acuminata* genome and found the number of *MaCOMT* gene family was the largest, while *MaC3H*s had only two members. *MaPAL*s retained the original members, and the number of *Ma4CL*s in lignin biosynthesis was significantly less than that of flavonoids. Segmental duplication existed in most gene families, except for *MaC3H*s, and tandem duplication was the main way to expand the number of *MaCOMT*s. Moreover, the expression profiles of lignin biosynthesis genes during fruit development, postharvest ripening stages and under various abiotic and biological stresses were investigated using available RNA-sequencing data to obtain fruit ripening and stress response candidate genes. Finally, a co-expression network of lignin biosynthesis genes was constructed by weighted gene co-expression network analysis to elucidate the lignin biosynthesis genes that might participate in lignin biosynthesis in banana during development and in response to stresses.

**Conclusion:**

This study systematically identified the lignin biosynthesis genes in the *Musa acuminata* genome, providing important candidate genes for further functional analysis. The identification of the major genes involved in lignin biosynthesis in banana provides the basis for the development of strategies to improve new banana varieties tolerant to biological and abiotic stresses with high yield and high quality.

## Introduction

Lignin is a complex and aromatic three-dimensional high molecular phenol polymer, which is widely distributed in nature, accounting for about 30% of the organic carbon in the biosphere (Campbell and Sederoff, 1996; Faraji et al., 2018). Lignin mainly exists in the secondary cell wall of all vascular plants and has many functions. For example, it can form an effective physical barrier against pathogens by cross-linking with the components of the cell wall (Lewis and Yamamoto, 1990). The lignin filled in the cellulose framework can also enhance the strength of plant cell wall and the bending resistance of stem, so as to improve the mechanical strength and lodging resistance of plant (Ahmad et al., 2020).

Lignin is mainly composed of three alcohol monomers, coniferyl, sinapyl and p-coumarin alcohol precursor, which are produced by coupling combination of oxidation of the p-hydroxycinnamic alcohol monomer, forming cinnamic acid through phenylalanine ammonia, and resulting in syringyl lignin (S), guaiacy lignin (G) and hyroxy-phenyl lignin (H) and other lignin monomer by a series of hydroxylation, methylation and reduction reaction, (Zhao and Dixon, 2011). These lignin monomers are finally dehydrogenated and polymerized into complex lignin complexes. The methoxylation level of S-, G- and H-type lignin monomers determines the lignin content and composition (Boerjan et al., 2003). In different plants, the content and composition of lignin are different. Generally, the lignins of pteriophyta and gymnosperm are mainly composed of G type lignin monomer and a small amount of H type lignin monomer. The dicotyledons in angiosperms are mainly composed of G-lignin monomer and S-lignin monomer, and the lignin in monocotyledons is a mixture of three monomers (Vanholme et al., 2010). Although the content and composition of lignin in different plants are different, the synthetic pathway is basically the same (Bonawitz and Chapple, 2010).

In most plants, there are mainly ten key enzyme gene families involved in lignin biosynthesis, including phenylalanine ammonia-lyase (PAL), cinnamate 4-hydroxylase (C4H), 4-coumarate: Co A ligase (4CL), hydroxycinnamoyl-Co A shikimate/quinate transferase (HCT), coumarate 3-hydroxylase (C3H), caffeoyl-Co A-O-methyltransferase (CcoAOMT), cinnamoyl Co A reductase (CCR), ferulate 5-hydroxylase (F5H), caffeic acid O-methyltransferase (COMT), cinnamyl alcohol dehydrogenase (CAD). Due to the increasing number of sequenced plant genomes, lignin biosynthesis genes have been identified in many plants, including *Arabidopsis* (Raes et al., 2003), *Populus trichocarpa* (Shi et al., 2010), Maize (Hu et al., 2009), Soybean (Baldoni et al., 2013), *Switchgrass* (Shen et al., 2013), *Eucalyptus grandis* (Carocha et al., 2015), Hard End Pear (Lu et al., 2015), Kenaf (Ryu et al., 2016), Pear (Cao et al., 2019), *Setaria viridis* (Ferreira et al., 2019), and Ramie (Tang et al., 2019).

Bananas (*Musa* spp.) are perennial monocotyledonous herbs that grow well in humid tropical and subtropical regions. In the banana pseudostem, the content of lignin in fiber is about 5-10%, lower than those of cellulose and hemicellulose (Komuraiah et al., 2014). Banana fruit is a popular tropical and subtropical fruit, and also is an important staple food for many people in tropical regions, rich in protein and carbohydrates (Aurore et al., 2009). As an important component of dietary fiber, lignin is an important index of quality of banana fruit and directly affects the fruit texture (Hamauzu et al., 1997; Happi Emaga et al., 2008). However, the expression pattern of lignin biosynthesis genes in banana fruit is rarely reported.

Cavendish banana is a triploid (AAA) banana cultivar formed by intraspecific hybridization in *Musa acuminata*. It is the most widely cultivated and commercialized variety (Perrier et al., 2011; Ploetz, 2015). After forming triploid, asexual propagation is the only reproductive mode of Cavendish banana, which leads to low environmental adaptability. Environmental stresses, such as drought (van Asten et al., 2011), low temperature (Kang et al., 2007), salt (Xu et al., 2014), and several devastating diseases (Heslop-Harrison and Schwarzacher, 2007) usually affect the yield and quality of the Cavendish banana fruits. Fusarium Wilt of Banana (FWB) is prevalent in main banana producing areas worldwide, which is seriously destructive for banana industry. It is a typical soil borne fungus disease caused by *Fusarium oxysporum* f. sp. *cubense* (Foc). Specifically, Cavendish banana is highly susceptible to Foc TR4 (Ploetz, 2006). Up to now, only a few genes have been cloned or used for expression analysis during Cavendish banana interacted with Foc TR4 (Wang et al., 2015; Wang et al., 2016). It is unclear about the roles of lignin biosynthesis gene in the process of fruit development, postharvest ripening and the response to stresses, especially those of the lignin biosynthesis genes involved in resistance to Foc TR4. This comprehensive study can increase our understanding of lignin biosynthesis genes of banana associated with fruit developmental and ripening processes and stresses responses and will establish a crucial foundation for future studies of genetic improvement in banana.

## Materials and methods

### Plant materials and treatments

Cavendish banana (*Musa acuminata* L. AAA group cv. Cavendish) fruits were harvested from the banana plantation of the Institute of Bioscience and Biotechnology (ITBB) of Chinese Academy of Tropical Agricultural Sciences (CATAS) (Wenchang, Hainan, 19.61N, 110.75E). The pulp of Cavendish banana fruit was harvested at 0, 20, and 80 DAF (day after flowering, DAF), which represented fruit developmental stages of budding, cutting flower, and harvest stages, respectively. During ripening stage, pulp of Cavendish banana fruit was harvested at 0, 8 and 14 DPH (day post-harvest, DPH) representing green, yellowish green, and yellow based on color of the fruit, respectively.

One month old Cavendish banana plantlets were grown under greenhouse conditions with 26°C and 80% humidity. Cavendish banana plantlets were irrigated with 300 mM NaCL and 200 mM mannitol for 7 days for salt and osmotic treatments, respectively. Cavendish banana plants were maintained at 4°C for 22 h for cold treatment. The leaves without main vein were harvested for analysis.

*Fusarium Wilt* of Banana (FWB) is prevalent in main banana producing areas worldwide, which is seriously destructive for banana industry. It is a typical soil borne fungus disease caused by *Fusarium oxysporum* f. sp. *cubense* (Foc). Cavendish banana is highly susceptible to Foc TR4. Giant Cavendish Tissue Culture Variants (GCTCV, GC) have acquired resistance to TR4 through somaclonal variation (Hwang and Ko, 2004). GCTCV-119 is the best Foc TR4-resistant alternative cultivar for the Cavendish (Ploetz, 2015). Variety Pahang (PH) is a wild banana belonging to subspecies *Musa acuminata* ssp. *malaccensis*, and highly resistant to Foc TR4 (Zhang et al., 2018). The fungus was grown on potato dextrose agar medium and incubated for 5-7 days at 28°C. The roots of 2-month old susceptible cultivar Cavendish banana, resistant cultivar GCTCV-119 and variety Pahang were dipped in Foc TR4 spore suspension of 1.0×10^6^ condia/mL, and the roots were harvested at 0 and 2 days post-infection (DPI) (Wang et al., 2012). All of the above samples were immediately frozen in liquid nitrogen and stored at -80°C.

### Identification of lignin biosynthesis genes in the *Musa acuminata* genome and phylogenetic analyses

To analyze the genome-wide lignin biosynthesis genes, we first downloaded all gene coding protein sequences of *Musa acuminata* genome (DH Pahang) from the Banana Genome Hub (https://banana-genome-hub.southgreen.fr) (Martin et al., 2016). We compared genes related to lignin biosynthesis genes in the *Musa acuminata* genome with the genes annotated in genomes of rice (http://rice.uga.edu/index.shtml) and *Arabidopsis* (http://www.arabidopsis.org/). In the PAL and HCT gene families, we also selected representative genes to draw the phylogenetic tree, including *EsPAL* (ATD50344.1) from *Eucalyptus saligna*, *JcPAL* (XP_012077001.1) from *Jatropha curcas*, *HbPAL* (XP_021684949.1) from *Hevea brasiliensis*, *PtHCT1* (XP_024452069.1) from *Populus trichocarpa*, *PtHCT2* (XP_006368492.1), *NtHCT* (NP_001312552.1) and *NtHQT*(NP_001312079.1) from *Nicotiana tabacum*, and *CcHCT* (ABO47805.1) from *Coffea canephora.* We retrieved protein sequences of these gene families for homologue-based searches with the criteria: similarity > 80% and coverage > 80%. Then, we confirmed the presence of the conserved domain within all the protein sequences in CDD (http://www.ncbi.nlm.nih.gov/cdd/) databases in NCBI and removed members without complete domain. The full-length lignin biosynthesis genes protein sequences from *Musa acuminata*, rice, *Arabidopsis* and other woody plants were aligned using ClustalW. Relationships were assessed using a Maximum Likelihood evolutionary tree with 1,000 bootstrap replicates and were created using MEGA 6.0 software (Tamura et al., 2013). The accession number of identified lignin biosynthesis genes was listed in **Supplementary Table 1**. The molecular weight and isoelectric points of the lignin biosynthesis genes were predicted from the ExPASy database (http://expasy.org/). The sequence logo for lignin biosynthesis genes domain was created by WebLogo server (http://weblogo.berkeley.edu/logo.cgi).

### Chromosome distribution and gene duplications

To determine the physical locations of lignin biosynthesis genes, the starting and ending positions of all lignin biosynthesis genes on each chromosome were obtained from the *Musa acuminata* genome database. MapInspect software was used to draw the images of the locations of the lignin biosynthesis genes (http://mapinspect.software.informer.com/). Tandem and segmental duplications were also identified according to the plant genome duplication database (Lee et al., 2012). Based on physical chromosomal location, homologous lignin biosynthesis genes on a single chromosome within 30 kb of each other were characterized as tandem duplication (Shiu and Bleecker, 2003; Du et al., 2013). Syntenic blocks were detected using MCSCAN (parameters: -a -e 1e-5 -s 5) (Wang et al., 2019), and all lignin biosynthesis genes located in the syntenic blocks were extracted. Circos (0.63) software was used to draw the images of the locations and synteny of the lignin biosynthesis genes (http://circos.ca/).

### RNA-seq data processing and expression profiling during fruit development, post-harvest ripening and stresses response

Total RNAs were extracted using Eastep Super Total RNA Extraction Kit (Promega, Beijing, China). Five μg of total RNA from each sample was converted to cDNA using GoScript Reverse Transcription Mix (Promega, Beijing, China), and constructed the cDNA libraries using TruSeq RNA Library Preparation Kit v2, and then were sequenced on an IIIumina HiSeq 2000 platform (San Diego, CA, USA) using the IIIumina RNA-seq protocol. Two biological replicates were used for each sample. Gene expression levels were calculated as Reads Per Kilobases per Million reads (RPKM) (Mortazavi et al., 2008). Differentially expressed genes were identified with the mean of read count from two replicates for each gene (fold change ≥ 2) (Audic and Claverie, 1997). MeV 4.9 and Java Treeview software constructed the heat-map of genes according to the manufacturer’s protocol. We analyzed the expression patterns of lignin biosynthesis genes during fruit development and postharvest ripening (0DAF, 20DAF, 80DAF-0DPH, 8DPH and 14DPH), and the response to abiotic (4°C for 22h, 300 mM NaCl for 7 days and 200 mM mannitol for 7 days) and biological (Roots of Cavendish banana, GCTCV-119 and Pahang varieties inoculated Foc TR4 after 2 days) stressors. All the data of RNA-seq had been uploaded to deposit in the CNSA (https://db.cngb.org/cnsa/) of CNGBdb with accession number CNP0000292. The accession numbers of all samples were listed in **Supplementary Table 2**.

### Weighted gene co-expression network analysis

Gene expression patterns for all identified TFs and the lignin biosynthesis genes from fruits, leaves and roots tissues were used to construct the co-expression network using WGCNA (version 1.47) within the R platform (version 3.2.2) (Langfelder and Horvath, 2008). Genes whose expression had not been detected in all tissues were removed in advance. Soft thresholds were set according to the scale-free topology criterion adopted by Zhang and Horvath (2005). An adjacency matrix was developed using the squared Euclidean distance values, and Pearson method was used to calculate the topological overlap matrix of unsigned network detection. Then, the co-expression coefficients greater than 0.50 between the target genes were selected. Finally, we extracted the co-expression network of lignin biosynthesis genes, and the network connections were visualized using cytoscape (Shannon et al., 2003). We enriched analysis of target genes using the Kyoto Encyclopedia of Genes and Genomes (KEGG) pathway.

### Yeast one-hybrid assay

Yeast one-hybrid screening was performed using MatchmakerTM Gold Yeast One-Hybrid Library Screening System (Clontech, Dalian, China). The bait fragment (the 1481 bp fragment of MaC3H2 promoter) was cloned into the pAbAi vector. The MaC3H2-AbAi and p53-AbAi were linearized and transformed into Y1HGold to make a bait-reporter strain, respectively. Transformants were initially screened on plates containing SD medium without Ura (SD/-Ura) and added with 0-1000 ng/ml aureobasidin A (AbA) for auto-activation analysis. Full-length coding sequences of *MaMYB1* (Ma02_t00290.1), *MaMYB2* (Ma06_t05680.1), *MaMYB3* (Ma11_t11940.1), *MaNAC1* (Ma07_t06080.1) and *MabHLH1* (Ma07_t28500.1) were cloned into the pGADT7 (AD) prey vector and transferred into the bait-reporter yeast strain, respectively. Transformed Y1HGold were incubated on SD medium with 300 ng/ml AbA and without leucine (SD-Leu+AbA300) at 28°C for 3 days to test the interaction. pGADT7-Rec (AD-Rec-P53) and p53-promoter fragments were co-transformed into Y1HGold as positive controls, while the AD-empty and MaC3H2-AbAi were used as negative controls. The primers used for yeast one-hybrid assay were listed in **Supplementary Table 3**.

### Dual luciferase reporter assay

Full-length coding sequences of the MaMYB1, MaMYB2, MaMYB3, MaNAC1 and MabHLH1 were inserted into the pGreen II 62-SK vector (SK), respectively, and the fragment of *MaC3H2* promoter was inserted into the pGreen II 0800-LUC vector. All the above constructs were transformed into *Agrobacterium tumefaciens* GV3101 using freeze-thaw method (Holsters et al., 1978). The dual luciferase assays were performed with *Nicotiana benthamiana* grown in green house for forty days. Agrobacterium cultures were prepared with infiltration buffer (10 mM MgCl_2_, 10 mM MES and 150 mM acetosyringone, pH5.6) to an OD_600_ of 0.8. Agrobacterium culture mixtures of TF genes (5 ml) and promoter (95 μL) were infiltrated into the abaxial side of *Nicotiana benthamiana* leaves. Leaves were collected after two days infiltration for luciferase (LUC) and Renilla luciferase (REN) activities analyses using Dual-Luciferase Reporter Assay System (Promega, USA, E1910) with Modulus Luminometers (Promega). Luciferase activity was analyzed in three independent experiments with six replications for each assay.

### Statistical analysis

Statistical analysis was performed using Student’s t-test. The experimental results obtained were expressed as the means ± standard deviation (SD). P values<0.05 were considered statistically significant (*), and P values<0.01 were considered highly statistically significant (**).

## Results

### Identification of lignin biosynthesis genes in *Musa acuminata* genome

In total, 104 genes belonging to ten key enzyme gene families, including eight *MaPALs*, five *MaC4Hs*, eighteen *Ma4CLs*, three *MaHCTs*, two *MaC3Hs*, seven *MaCCoAOMTs*, twenty-four *MaCCRs*, four *MaF5Hs*, twenty-four *MaCOMTs* and ten *MaCADs* were identified in the *Musa acuminata* genome. In addition, we found 31 genes identified as ‘like’ genes (candidate genes excluded from *bona fide* clade), including 9 from *Ma4CLs*, 21 from *MaCCRs* and 1 from *MaCOMTs* (**Supplementary Table 1**). Gene names were based on the location of chromosomes. The gene ID, position, and protein physicochemical properties of all genes were shown in **Supplementary Table 1**.

**Figure 1:**
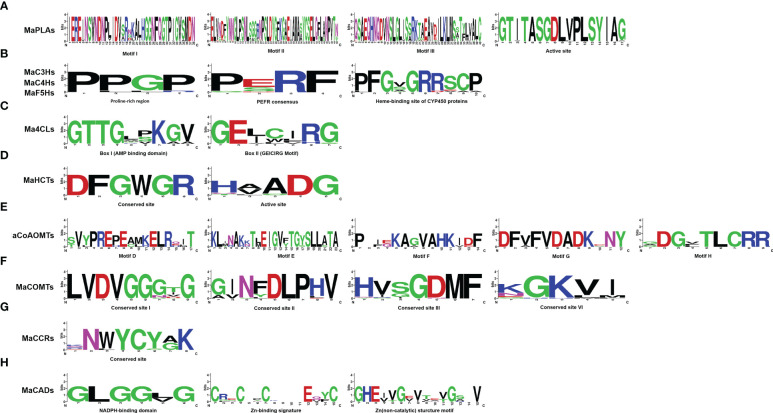
Sequence logo of the lignin biosynthesis proteins motifs. The height of each amino acid represented the relative frequency of the amino acid at that position. **(A)** Phenylalanine ammonialyase(PAL), **(B)** coumarate 3-hydroxylase (C3H), cinnamate 4-hydroxylase (C4H) and ferulate 5-hydroxylase (F5H), **(C)** 4-coumarate: CoA ligase (4CL), **(D)** hydroxycinnamoyl-CoA shikimate/quinate transferase (HCT), **(E)** caffeoyl-CoA-O-methyltransferase (CcoAOMT), **(F)** caffeic acid Omethyltransferase (COMT), **(G)** cinnamoyl CoA reductase (CCR), **(H)** cinnamyl alcohol dehydrogenase (CAD).

In conservative domain analysis, we mainly compared the lignin biosynthesis genes from banana with genes from *Arabidopsis*. We found all genes contained the core conserved domains belonging to the features of gene family in the protein sequence. MaPALs contained the conserved motifs (GTITASGDLUPLSYIAG) (**Figure 1A**). MaC3Hs, MaC4Hs and MaF5Hs had cytochrome P450 conserved domains, such as proline rich region (PPGPKGLP), PERF conserved motif and heme-binding motif of CYP450 proteins (PFGSGRRSCP) (**Figure 1B**). In Ma4CLs, all of them contained conservative Box I (SSGTTGLPKGV) and Box II (GEICVRS) in the protein sequence (**Figure 1C**). We found *MaHCT*s genes contained the conserved active motif (HXXXDG) and conservative motif (DFGWGR) of acetyltransferase gene family (**Figure 1D**). Generally, the amino acid sequence of CCoAOMT gene family contains 8 conserved motifs, in which motif D, motif E, motif F, motif G and motif H are unique to the CCoAOMT family and are as the tag sequences (Zhao et al., 2004). We found all MaCCoAOMTs contained motif D, motif E, motif F, motif G and motif H (**Figure 1E**). In these sequences of COMT proteins, there were five amino acid conserved elements in proper order from the N-terminal to the C-terminal of the amino acid sequence (conservative motif 1: LVDGGGxG, conservative motif II: GINFDLPHV, conservative motif III: EHVGGDMF and conservative motif VI: NGKVI) (Ibrahim et al., 1998). In *Musa acuminata* genome, we found that 23 COMT genes had the above conserved domains (**Figure 1F**). Three *bona fide* MaCCRs (MaCCR1, MaCCR2 and MaCCR3) were identified to contain the conserved sequence (KNWYCYGK) belonging to CCR gene family (**Figure 1G**). Ten MaCAD proteins contained binding domain for the structurally important zinc (CX2CX2CX7C), NADPH (GxGGVG) and catalytic Zn^2+^ binding motif (GHExxGxxxxxGxxV), which was the signature sequence of CAD genes (**Figure 1H**). The above results show that all 104 lignin biosynthesis genes have typical conserved domains of their respective gene families.

### Phylogenetic analysis of lignin biosynthesis genes from *Musa acuminata* genome

Analyses of the evolutionary relationships of lignin synthetase genes from *Musa acuminata*, *Arabidopsis*, rice, and other plants have been functionally verified. The phylogenetic tree was analyzed by MEGA6 using the method with 1000 bootstrap replicates and the evolutionary distances were calculated using the Poisson correction method.

**Figure 2:**
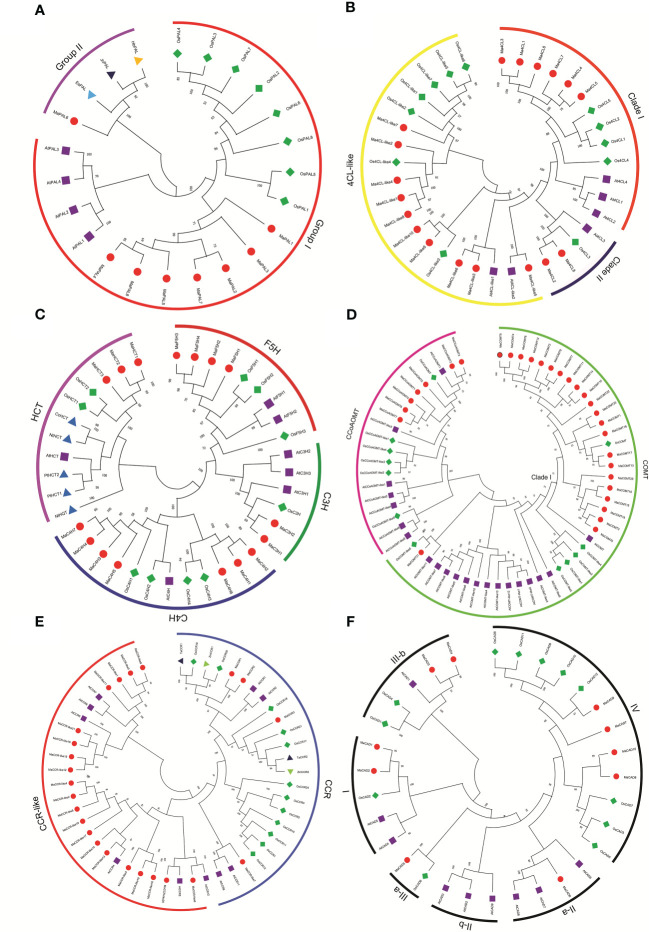
Phylogenetic relationship of lignin biosynthesis genes in *Musa acuminata* (red dot), *Arabidopsis* (purple square), rice (green diamond), and other plants (triangle). The Maximum Likelihood (ML) tree was drawn using MEGA 6.0 with 1000 bootstrap replicates. **(A)** Phenylalanine ammonialyase(PAL), **(B)** 4-coumarate: CoA ligase (4CL), **(C)** hydroxycinnamoyl-CoA shikimate/quinate transferase (HCT), coumarate 3-hydroxylase (C3H), cinnamate 4-hydroxylase (C4H) and ferulate 5-hydroxylase (F5H), **(D)** caffeic acid Omethyltransferase (COMT) and caffeoyl-CoA-O-methyltransferase (CcoAOMT), **(E)** cinnamoyl CoA reductase (CCR), **(F)** cinnamyl alcohol dehydrogenase (CAD).

MaPAL family can be clearly divided into two groups. MaPAL1, MaPAL2, MaPAL3, MaPAL4, MaPAL5, MaPAL7 and MaPAL8 were clustered together with all PALs from *Arabidopsis* and rice. MaPAL6 was placed near the PALs from woody plants, such as EsPAL, JcPAL and HbPAL, indicating that the evolution of MaPALs is more conservative, MaPAL6 may have different origins from the other seven MaPALs (**Figure 2A**). In plants, 4CL can be divided into three clades, clade I is thought to be related to lignin synthesis, and clade II 4CL is thought to be related to flavonoid synthesis (Wei and Wang, 2004). The Ma4CLs can be divided into three clades, Ma4CL1, Ma4CL3, Ma4CL4, Ma4CL5, Ma4CL6, Ma4CL7 were located in clade I, Ma4CL2 and Ma4CL8 were located in clade II, and others were located in 4CL-like clade. Moreover, we also found that the Ma4CLs were often closer to Os4CLs and far away from At4CLs (**Figure 2B**). It is suggested that Ma4CL11, Ma4CL14, Ma4CL6 and Ma4CL7 may be related to lignin synthesis and formed after the differentiation of monocotyledons and dicotyledons. MaC4Hs can be divided into two clades: MaC4H, MaC4H3, MaC4H4, MaC4H5, MaC4H7, AtC4H, OsC4H1 and OsC4H2 which were located in same clade, while MaC4H1, MaC4H2, MaC4H6, OsC4H3 and OsC4H4 were located in the same clade (**Figure 2C**). MaC3Hs were more closely related to OsC3H than AtC3H1 and no member was close to AtC3H2 and AtC3H3 (**Figure 2C**). Those results indicate that the evolution of MaC4Hs and MaC3Hs may be formed after the differentiation of monocotyledons and dicotyledons. The members of MaF5Hs were clustered with the AtF5Hs, and far from OsF5Hs (**Figure 2C**), indicating that the differentiation of F5H gene is formed before the differentiation of monocotyledons and dicotyledons. Three MaHCTs were distributed in the same clade with OsHCT1 and OsHCT2, but far away from the HCTs from *Arabidopsis thaliana*, *Populus trichocarpa* (PtHCT1, PtHCT2), *Nicotiana tabacum* (NtHCT and NtHQT) and *Coffea canephora* (CcHCT), indicating that MaHCTs may be formed after differentiation of monocotyledons and dicotyledons (**Figure 2C**). CCoAOMTs can be mainly divided into two clades (Rakoczy et al., 2018). All seven MaCCoAOMTs were distributed in the same clade with OsCCoAOMT and AtCCoAOMT1 (**Figure 2D**). Similarly, all the MaCOMTs located with AtOMT1 and OsCOMT1 belonged to the Clade I, and only MaCOMT21 close to AtOMT-like11 and OsCOMT-like5 belonged to COMT-like (**Figure 2D**). *Bona fide* CCR clade and CCR-like clade were contained in CCR gene family. In particular, MaCCR1 and MaCCR2 were closely related to TaCCR1, ZmCCR1, AtCCR1 and AtCCR2. MaCCR3 was grouped with ZmCCR2, TaCCR2, and PvCCR2 (**Figure 2E**). Thirty-one CADs were clearly divided into three classes (class I, class II and class III). MaCAD1 and MaCAD2 were located in class I (*bona fide* CAD clade) with AtCAD4, AtCAD5 and OsCAD2. MaCAD5, MaCAD6, MaCAD7, MaCAD8, MaCAD9 and MaCAD10 were distributed in class II. MaCAD3 and MaCAD4 were located in class III with AtCAD1, OsCAD1 and OsCAD4 (**Figure 2F**).

### Chromosomal localization and gene duplication of lignin biosynthesis genes from *Musa acuminata* genome

**Figure 3:**
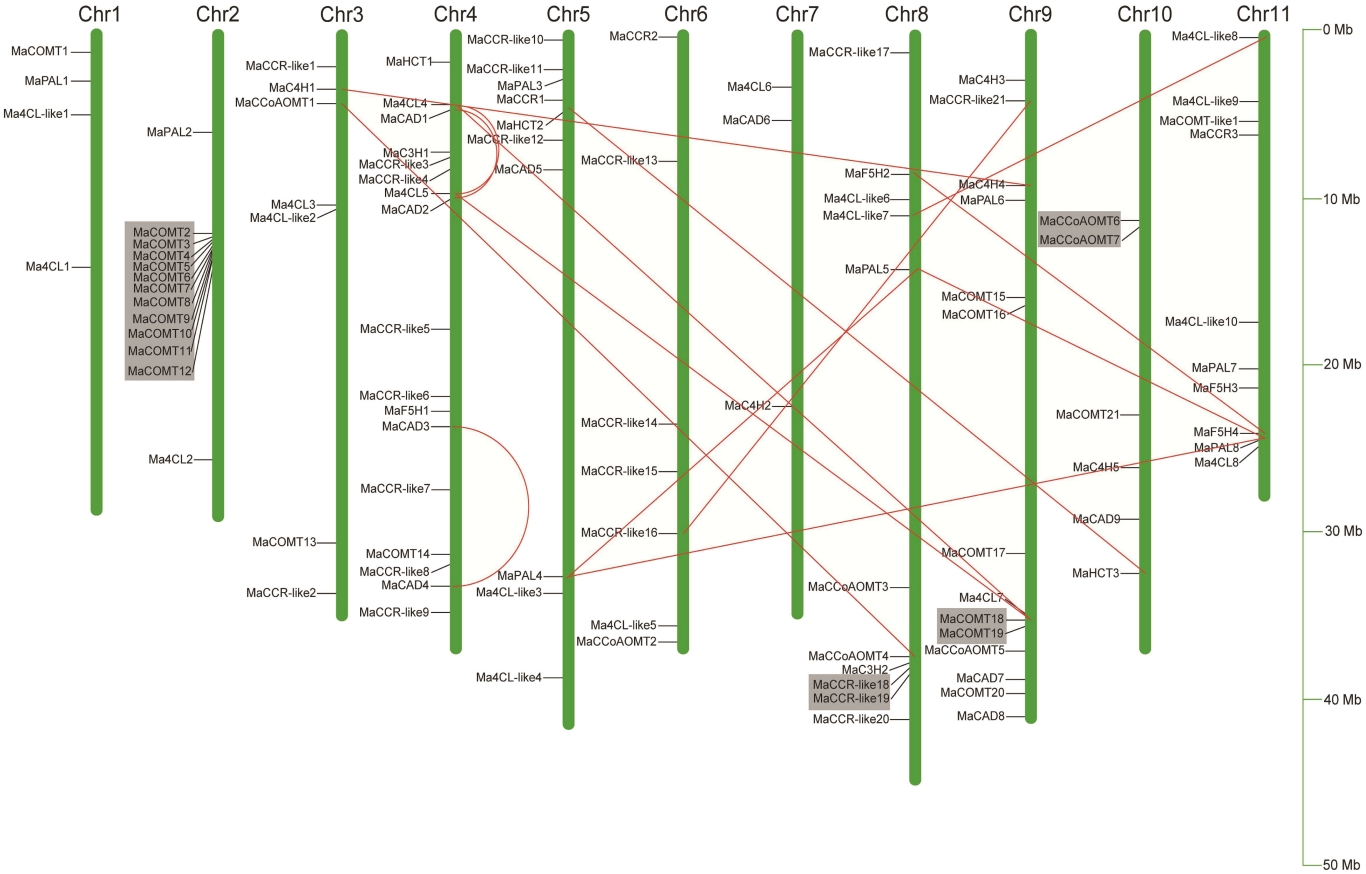
Localization of 102 lignin biosynthesis genes on eleven *Musa acuminata* genome chromosomes. The chromosomes were numbered between 1 to 11 and shown at the top of each chromosomes (Chr represented as bars). Chromosomal distances were given in Mb. Red line indicated segmental duplicated genes, and gray area indicated tandem duplicated genes.

In order to determine the distribution of lignin biosynthesis genes on the chromosome, the relative positions of 101 genes on the chromosome were identified according to their physical positions in the *Musa acuminata* genome database. Ma00_t01670.1, Ma00_t02580.1 and Ma00_t03560.1 were not anchored on the chromosome and were named *MaCOMT22*, *MaCOMT23* and *MaCAD10*, respectively. As shown in **Figure 3**, although each of the 11 banana chromosomes contained lignin biosynthesis genes, the distribution appeared to be uneven. There were 17 members on chromosome 4 from 7 different gene families (*MaHCTs*, *Ma4CLs*, *MaCADs*, *MaC3Hs*, *MaCCRs*, *MaF5Hs* and *MaCOMTs*). There were 14 members on chromosome 9 from six families (*Ma4CLs*, *MaC4Hs*, *MaCADs*, *MaCCoAOMTs*, *MaCOMTs* and *MaPALs*), while only three genes on chromosome 3 were from three families (*Ma4CL6*, *MaCAD6* and *MaC4H2*). In the multi member gene families, eighteen *Ma4CLs* were widely distributed on the 10 chromosomes except for Chr10. Twenty-four *MaCCRs* were distributed on 6 chromosomes as follows Chr3, Chr4, Chr5, Chr6, Chr8 and Chr9. Most of *MaCOMTs* were only distributed on Chr2 and Chr9.

Segmental duplication, tandem duplication and retrotransposition are known to be key factors driving gene family expansion (Hurles, 2004; Freeling, 2009). In our study, 11 colinear genes pairs from nine gene families except for *MaC3Hs* were identified to be located in the synthetic blocks and belonged to segmental duplication (**Figure 3** and **Supplementary Table 4**). It is worth noting that there were four *MaCADs* (*MaCAD1*, *MaCAD2*, *MaCAD3* and *MaCAD4*) with segmental duplication located in Block4_chr04, Block8_chr01 and Block8_chr04, indicating that the segmental duplication plays important roles in the expansion of *MaCADs*. We also found four tandem duplication gene clusters on Chr2, Chr8, Chr9 and Chr10. The number of gene clusters on chromosome 2 was the largest, containing 11 genes, and all of them were from *MaCOMT* gene family (**Figure 3**). We did not find the member belonging to retrotransposition among the 104 lignin biosynthesis genes. Based on our analysis, we propose that the expansion of lignin biosynthesis genes may be mainly *via* segment and tandem duplication during the *Musa acuminata* genome evolutionary processes.

### Expression profile of lignin biosynthesis genes during Cavendish fruit development and the postharvest ripening stage

**Figure 4:**
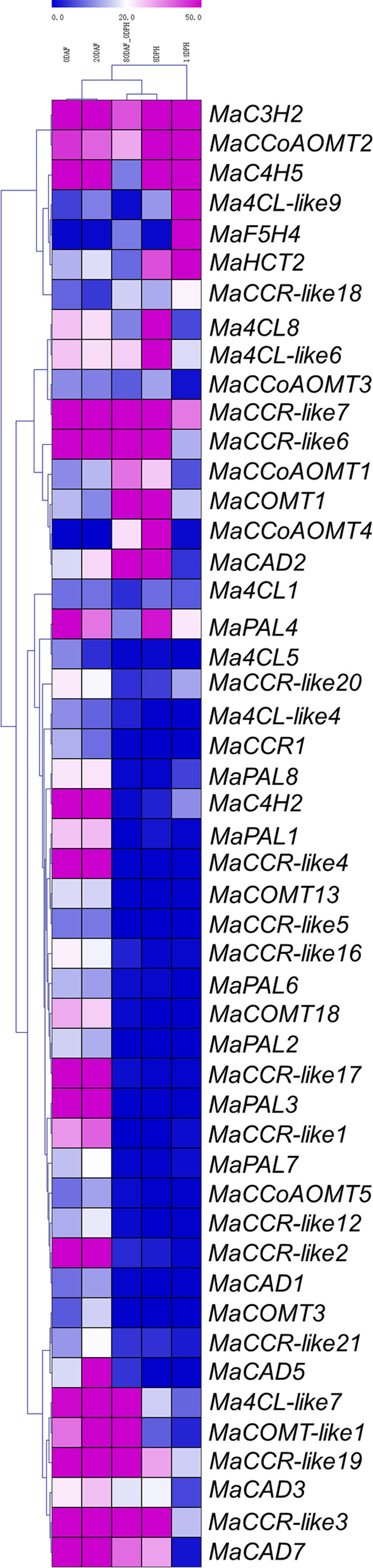
Expression patterns of lignin biosynthesis genes in different stages of fruit development and ripening. The heatmap with dendrogram was created based on the RPKM value of the lignin biosynthesis genes. Differences in gene expression changes were shown in color as the scale.

To analyze the expression profiles of lignin biosynthesis genes, RNA-Seq data were derived from the fruit development and ripening stages (**Supplementary Table 2**). We deleted 56 lignin biosynthesis genes with RPMK values less than 5 in 0DAF, 20DAF, 80DAF_0DPH, 8DPH and 14DPH. There were 25 and 26 lignin biosynthesis genes highly expressed at 0 DAF and 20 DAF (RPKM > 20), contributing about 24.04% and 25.00% of the total, respectively. In 0DAF and 20 DAF, the expression levels of *MaC3H2*, *MaC4H2*, *MaC4H5*, *MaCAD7*, *MaCCR-like17*, *MaCCR-like19*, *MaCCR-like2*, *MaCCR-like3*, *MaCCR-like4*, *MaCCR-like6*, *MaCCR-like7* and *MaPAL3* were more than 50 (RPKM>50). However, in 80 DAF-0 DPH, 8 DPH and 14 DPH, only 14, 16 and 10 genes were highly expressed (RPKM>20), respectively (**Figure 4**). The number of highly expressed genes during postharvest ripening was significantly less than that during fruit development. These results indicate that the lignin biosynthesis genes are expressed actively in the fruit development, which is closely related to the growth and development of banana fruit. It is worth noting that *MaC3H* gene family contained two copies (*MaC3H1* and *MaC3H2*) (**Figure 3**). During fruit development and ripening, we found that *MaC3H1* was not expressed in 11 transcriptome data and checked by RT-PCR (results not shown), while *MaC3H2* was constitutively expressed with high RPKM value (RPKM>40) (**Supplementary Table 5**), showing that only *MaC3H2* may have biological functions. We also found that the expression levels of *Ma4CL8*, *MaPAL4*, *MaC4H5*, *Ma4CL-like6*, *MaCCoAOMT2*, *MaCAD3*, *MaHCT2* and *MaCAD2* were increased gradually in 80DAF-0DPH, 8DPH and 14DPH, and some genes such as Ma4CL-like9, *MaHCT2*, *MaF5H4* and *MaC4H5* reached the highest level in 14DPH (RPKM>190) (**Figure 4**). The above results suggest that these lignin biosynthesis genes may play roles in the lignin synthesis during the postharvest process of banana fruit and participate in the quality formation.

### The expression level of lignin biosynthesis genes in the Cavendish banana plantlets in response to osmotic, salt, and cold treatments

**Figure 5:**
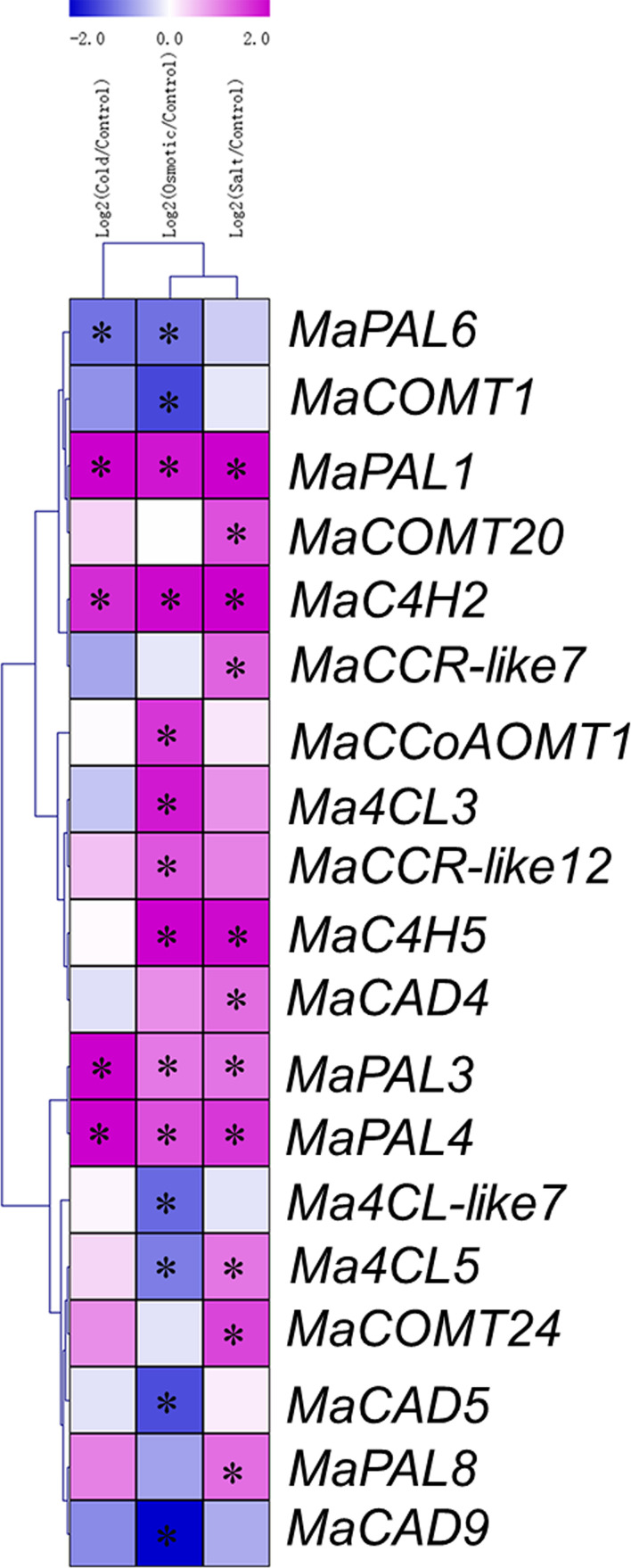
Expression patterns of lignin synthesis genes in response to cold, osmotic, and salt treatments in banana. The Log2-based fold change was used to create the heatmap. Differences in gene expression changes were shown in color as the scale. * Significant difference at *P* < 0.01.

To analyze the expression profiles of lignin biosynthesis genes, RNA-Seq data were derived from Cavendish banana plantlets in response to osmotic, salt and cold treatments. To present the differentially expressed genes visually and exactly, we filtered out the genes with RPKM values less than 5 in the control or treatments. Cold treatment (4°C) caused the fifteen lignin biosynthesis genes were differentially expressed (log2 Ratio Cold/Control>1), among which eleven were up-regulated and four down-regulated (**Figure 5** and **Supplementary Table 6**). Treatment with drought (200 mM mannitol) caused the eighteen genes were found to be differentially expressed (log2 Ratio Osmotic/Control > 1), among which nine were up-regulated and nine down-regulated (**Figure 5** and **Supplementary Table 6**). Treatment with salt (300 mM NaCl) caused fourteen members were differentially expressed (log2 Ratio Salt/Control > 1), among which thirteen were up-regulated and one was down regulated (**Figure 5** and **Supplementary Table 6**). It is worth noting that *MaPAL1*, *MaPAL3*, *MaPAL4*, *MaPAL7*, *MaC4H2* and *MaCCR9* were up-regulated during the drought, salt, and cold stresses. We found that forty-one genes were significantly differentially expressed under cold, drought and salt stresses, but nine genes were only involved in one of the abiotic stresses (**Figure 5**). Therefore, most of the genes were not differentially expressed in the responses to above stresses. For example, we did not find that *bona fide MaCCRs* was involved in the process of the responses to above abiotic stresses. Although several *MaCCR-likes* were found to be up-regulated under low temperature, drought and salt stresses, the function of *MaCCR-like* was not clear. Similarly, we also found that only one member of *MaCADs* was up-regulated under salt stress, other eight *MaCADs* were not differentially expressed during the responses to abiotic stresses. Moreover, in phylogenetic tree, the differentially expressed *MaCAD4* was closely related to *AtCAD1*, which played a partial role in the synthesis of lignin in *Arabidopsis* (Eudes et al., 2006). *MaCAD2* and *MaCAD3*, belonging to the group I (the *bona fide* CAD clade) (**Figure 2**), were not differentially expressed under the drought, salt and cold stresses (**Figure 5**). These results indicate that most of lignin synthesis genes are not involved in banana response to stresses, which may be one of the possible reasons for banana being more sensitive to abiotic stresses.

### The expression level of lignin biosynthesis genes during the banana plantlets interacting with Foc TR4

**Figure 6:**
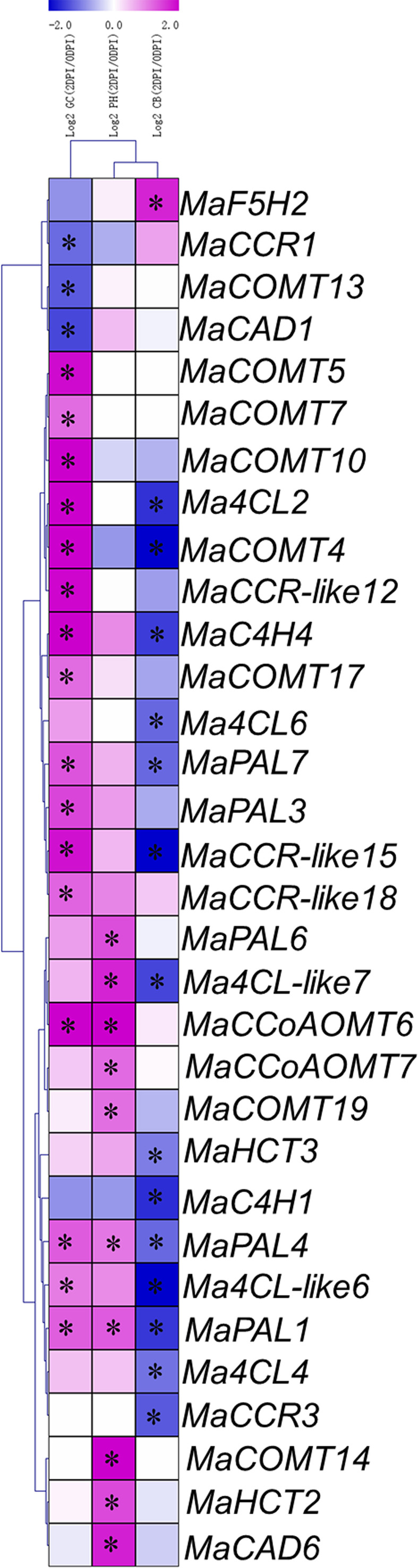
Expression patterns of lignin biosynthesis genes in Cavendish banana (CB), GCTCV-119 (GC) and Pahang (PH) inoculated with Foc TR4. The Log2-based fold change was used to create the heatmap. Differences in gene expression changes were shown in color as the scale. * Significant difference at *P* < 0.01.

The expression pattern of a gene is generally correlated with its function; hence, we analyzed the expression patterns of the lignin biosynthesis genes in susceptible and resistant varieties responding to Foc TR4 infection. To present the differentially expressed genes visually and exactly, we filtered out the genes with RPKM values less than 5 in the 0 DPI or 2 DPI. In variety Cavendish, there were only 11 differentially expressed genes (marked with black stars), of which only one was up-regulated and 10 down-regulated (**Figure 6** and **Supplementary Table 7**). The results showed that lignin synthesis genes were not activated in susceptible varieties. In variety Pahang, there were 12 differentially expressed genes from seven gene families, all of which were up-regulated (**Figure 6** and **Supplementary Table 7**), indicating that lignin biosynthesis genes were activated in banana responding to Foc TR4 infection. In variety GCTCV-119, we found 20 differentially expressed genes, 17 of which were up-regulated and 3 down-regulated (**Figure 6** and **Supplementary Table 7**), indicating that most of the differentially expressed genes were activated. It is noteworthy that we found that the differentially expressed genes between Pahang and GCTCV-119 were not consistent, indicating that ploidy still has a great impact on the banana resistance to Foc TR4 infection. In general, our results indicate that these differentially expressed lignin biosynthesis genes are involved in banana resistance to Foc TR4 infection.

**Figure 7:**
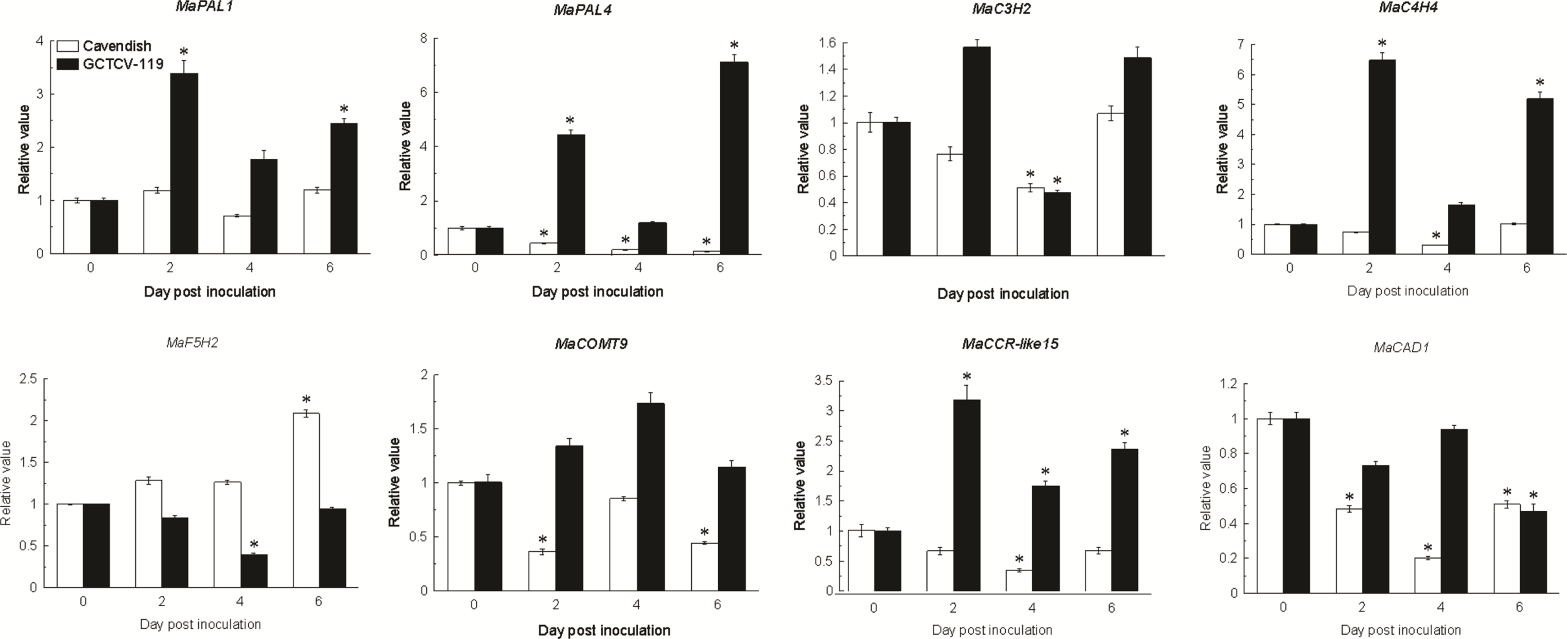
Expression patterns of lignin biosynthesis genes involving in plant-pathogen interaction pathway in Ba Xijiao and GCTCV-119 inoculated with Foc TR4 by qRT-PCR. The results are presented as differential relative transcript abundance. The data represent the mean ± standard deviation (SD) of three replicates. The y-axis shows the transcript fold-change relative to that in the control (0 DPI). * Significantly different from the control (0 DPI) at *P* < 0.01, respectively.

Based on RNA-seq data, 8 differentially expressed lignin biosynthesis genes were selected for RT-qPCR. The results showed that the expression patterns of the selected genes had the same trend and consistent results between RNA-seq data and RT-qPCR data (**Figure 7**).

### Weighted gene co-expression network of lignin biosynthesis genes

**Figure 8:**
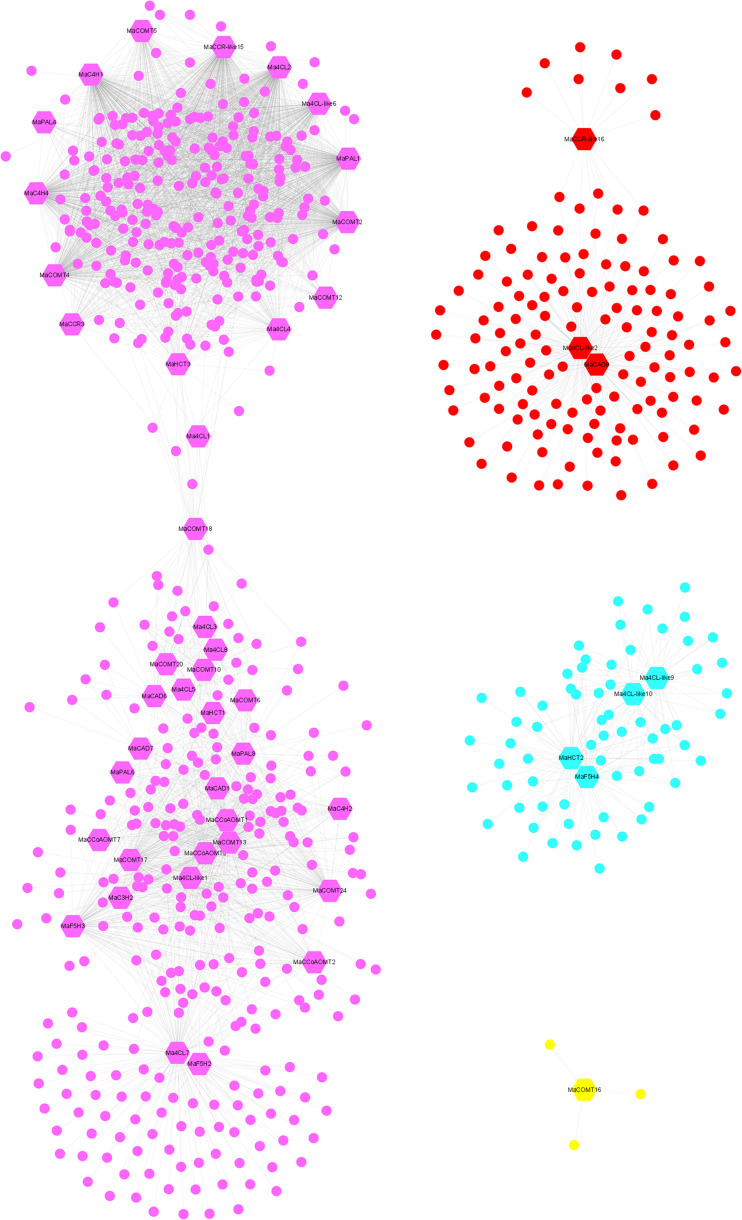
Co-expression network of banana generated using the lignin biosynthesis genes as guides. The network comprised 725 TF genes (nodes); the hexagon represented lignin biosynthesis genes.

To explore the functions, 104 lignin biosynthesis genes were selected as ‘guide genes’ to seek co-expressed genes using an RNA-Seq dataset from 11 different transcriptomes, including fruit development and ripening stages, banana plantlets response to osmotic, salt, and cold treatment, and banana roots inoculated with Foc TR4 (**Supplementary Table 2**). A total of 725 TFs were as the ‘target nodes’, whose expression patterns were closely correlated with 51 lignin biosynthesis genes from ten gene families, which were identified with weighted values larger than 0.5 (**Supplementary Table 6**). Following visualization using Cytoscape (Otasek et al., 2019), the co-expression network of lignin biosynthesis genes was divided into four modules. Module I-IV (purple, red, light blue and yellow, respectively) contained 37, 3, 4, 1 and 1 lignin biosynthesis genes, respectively (**Figure 8**). The correlated TFs were in 68 different type TF classes, such as MYB, ERF, basic helix-loop-helix (bHLH), WRKY, C2H2, NAC, and LOB (**Supplementary Table 8**).

### Verifying the interaction between promoter of *MaC3H2* and transcription factors in WGCNA using yeast one-hybrid

The -2000bp upstream sequence of *MaC3H2* was obtained by RT-PCR based on the sequence of *Musa acuminata* genome, the 1481bp promoter sequence was obtained by transcriptional active site prediction, and the main regulatory elements of *MaC3H2* promoter were predicted online by plantcare website. We found that there was conserved AC element (AC-I element, -1378bp) and MBS and MRE elements (-32 and -1066bp) bounding to MYB TF in the *MaC3H2* promoter sequence (**Supplementary Table 3**). The results show that *MaC3H2* may be closely related to lignin synthesis in banana.

**Figure 9:**
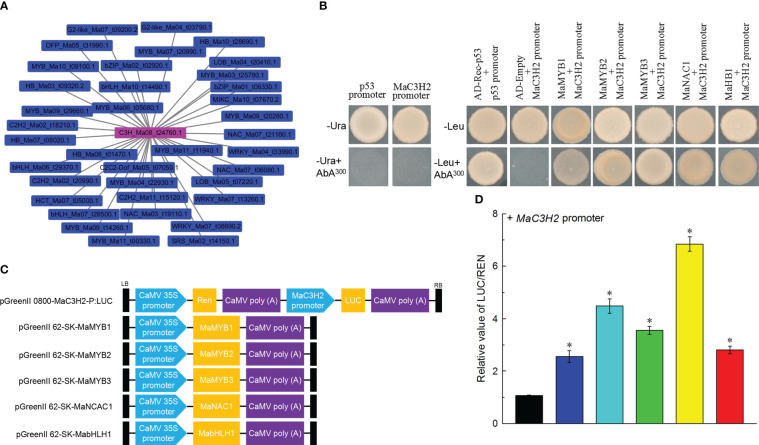
Identification of upstream regulatory transcription factors of MaC3H2. **(A)** Co-expression network of MaC3H2 by WGCNA. **(B)** Yeast one-hybrid assay of MaMYB1, MaMYB2, MaMYB3, MaNAC1 and MabHLH1 bind with MaC3H2 promoter. **(C)** Schematic diagrams of the effector and reporter constructs used for the dual LUC assay. **(D)** MaMYB1, MaMYB2, MaMYB3, MaNAC1 and MabHLH1 activate the MaC3H2 promoter in dual-luciferase assays. * Significant difference at *P* < 0.01.

We extracted the co-expression network of *MaC3H2* and found 36 transcription factors from 13 gene families were contained in the network, and *MaMYBs* had the largest number (10 *MaMYBs*) (**Figure 9A**). We further identified whether the *MaC3H2* promoter had a relationship with these transcription factors. Five transcription factors including *MaMYB1* (Ma03_t25780.1), *MaMYB2* (Ma09_t20280.1), *MaMYB3* (Ma11_t11940.1), *MaNAC1* (Ma07_t06080.1) and *MabHLH1* (Ma10_t14490.1) were verified by yeast one hybridization. Using the pGADT7-MaMYB1, pGADT7-MaMYB2, pGADT7-MaMYB3, pGADT7-MaNAC1, pGADT7-MabHLH1 vectors as effectors and using the pAbAi vector carrying *MaC3H2* promoter as a reporter, the Y1HGold yeast co-transformed with *MaC3H2* promoter-AbAi and pGADT7-MaMYB1, pGADT7-MaMYB2, pGADT7-MaMYB3, pGADT7-MaNAC1, pGADT7-MabHLH1 could grow normally on the SD/-Leu medium containing 300 ng/mL AbA, respectively, whereas the yeast carrying MaC3H2 promoter-AbAi and pGADT7 could not grow (**Figure 9B**), which proved that *MaMYB1*, *MaMYB2*, *MaMYB3*, *MaNAC1* and *MabHLH1* can definitely bind with the sequence of the *MaC3H2* promoter, respectively.

In the dual luciferase assay, the *MaC3H2* promoter-driven luciferase reporter and CaMV35S-driven *MaMYB1*, *MaMYB2*, *MaMYB3*, *MaNAC1* and *MabHLH1* effector were separately constructed and then co-infected tobacco leaf epidermal cells by *Agrobacterium-mediated* transformation (**Figure 9C**). *MaMYB1*, *MaMYB2*, *MaMYB3*, *MaNAC1* and *MabHLH1* were found to activate the activity of the *MaC3H2* promoter with a LUC/REN ratio higher than 2.12, 4.57, 3.81, 6.41 and 3.04-fold of empty vector (pGreen II 62-SK/pGreen II 0800-LUC), respectively, indicating that *MaMYB1*, *MaMYB2*, *MaMYB3*, *MaNAC1* and *MabHLH1* are activative effectors of *MaC3H2* gene expression (**Figure 9D**).

## Discussion

Lignin is an important product of phenylpropane metabolism pathway and an important component of plant cell wall, playing crucial roles in maintaining normal development, enhancing overall mechanical strength and increasing stress resistance of plants (Zhao and Dixon, 2011). The systematic identification of the enzyme genes in the lignin biosynthesis pathway in *Musa acuminata* genome can provide important references for improving the content and composition of lignin to obtain ideal new varieties with good quality and strong resistance for banana industry. Generally, there are usually 10 enzyme gene families involved in lignin biosynthesis (Xu et al., 2009). So far, many important plants have identified genes for lignin biosynthesis enzyme, 56 genes encoding *bona fide* lignin biosynthesis enzymes in *Setaria viridis* genome (Ferreira et al., 2019), 37 genes in *Eucalyptus grandis* (Carocha et al., 2015), 34 genes in *Arabidopsis* (Raes et al., 2003), 35 genes in *Pyrus bretschneideri* (Cao et al., 2019). In this study, we totally identified 104 genes belonging to ten lignin biosynthesis gene families in *Musa acuminata* genome, and 73 genes encoding *bona fide* enzymes, which have been the highest number of *bona fide* lignin biosynthesis genes detected, to date. The characteristics of the number of encoding amino acids, isoelectric point, and molecular weight of the lignin biosynthesis genes are similar to previous results in *Arabidopsis* and other plants (Raes et al., 2003; Carocha et al., 2015; Cao et al., 2019).

Gene duplication is universal across all organisms and plays a key role in driving the evolution of genomes and genetic systems, and is closely related to translocation, insertion, inversion, deletion and duplication of small fragments, chromosome rearrangement and fusion after genome-wide duplication events (Simillion et al., 2004; Navajas-Pérez and Paterson, 2009). The *Musa acuminata* genome underwent three rounds of WGD (α/β/γ), resulting in a large number of collinear blocks in the *Musa acuminata* genome (D'Hont et al., 2012), and showed that the chromosomes of *Musa acuminata* genome had experienced multiple rounds of genome-wide duplication events. At the gene family level, we found that nine lignin biosynthesis gene from *Musa acuminata* genome underwent tandem or segmental duplication events except for *MaC3Hs* which have only two members and may be the reason leading the *Musa acuminata* genome has more gene members than those in *Arabidopsis* and rice. We found the number of *MaC4Hs*, *MaF5Hs*, *MaC3Hs*, *MaHCTs* and *MaCCoAOMTs* gene families was relatively small, which was not different from that in *Arabidopsis* and rice (**Figure 2**).

In the phylogenetic tree, MaPAL6 was divided into a subfamily with PALs of other woody plants (**Figure 2**). This is similar to the results of other species (Shi et al., 2010). 4CL gene family usually exists as a small gene family (Gui et al., 2011; Sun et al., 2013). There are four 4CL genes in *Arabidopsis* and five 4CL genes in rice, maize and *Populus* (Sun et al., 2013; Li et al., 2015; Rao et al., 2015; Xiong et al., 2019). In our results, we found eight 4CL genes were in *Musa acuminata* genome. Phylogenetic analysis show that 4CL in dicotyledons can be divided into two types. In type I, 4CL was mainly involved in lignin biosynthesis, while in type II, 4CL was often involved in flavonoids biosynthesis (Li et al., 2015). In *Musa acuminata* genome, we found six Ma4CLs in type I and two Ma4CLs in type II (**Figure 2**), indicating that the number of Ma4CL involved in lignin biosynthesis is more than that involved in flavonoid biosynthesis. During fruit development and postharvest ripening stages, we found that the RPKM value of *Ma4CL8* involved in flavonoid synthesis was significantly higher than that of *Ma4CL1*, *Ma4CL5* and *Ma4CL6* involved in lignin biosynthesis, and showed that the metabolism of flavonoids may be stronger than that of lignin in banana fruit (**Figure 4**). We also found that *Ma4CL3* and *Ma4CL5* involved in lignin synthesis were significantly differentially expressed in osmotic and salt stresses, while the *Ma4CL2* and *Ma4CL8* involved in flavonoid synthesis were not differentially expressed in low temperature, drought and salt stresses (**Figure 5**), indicating that *Ma4CLs* may be involved in Cavendish banana responding to abiotic stresses.

In plants, the COMT gene family usually comprises multiple members. For example, there are seven COMT genes in *Eucalyptus grandis* (Carocha et al., 2015), 25 in *Populus trichocarpa* (Shi et al., 2010) and 18 in *Arabidopsis thaliana* and 27 in *Vitis vinifera* (Liu et al., 2016). In our results, 24 MaCOMTs were detected in *Musa acuminata* genome. All the MaCOMTs were near by the *AtOMT1* (At5g54160.1) and *OsCOMT1* (Os08G06100) and located in clade I (**Figure 2D**). *AtOMT1* and *OsCOMT1* have been identified to participate in lignin biosynthesis in *Arabidopsis* and rice (Goujon et al., 2003; Raes et al., 2003; Vanholme et al., 2012; Dalmais et al., 2013; Koshiba et al., 2013). These results suggest that the MaCOMTs from *Musa acuminata* genome may have the ability to participate in lignin biosynthesis. In *Arabidopsis*, AtCCR1 is involved in constitutive lignification (Lauvergeat et al., 2001). ZmCCR1 and TaCCR1 may participate in developmental lignin deposition in secondary cell walls (Tamasloukht et al., 2011). TaCCR2 is actively undergoing lignification and involved in both G and S lignin synthesis (Ma and Tian, 2005). In phylogenetic tree, we found MaCCR1 and MaCCR2 were near by the ZmCCR1, TaCCR1, OsCCR19 and OsCCR20, and in this clade we also found AtCCR1. MaCCR3 was grouped with TaCCR2 (**Figure 2E**), suggesting that MaCCR1, MaCCR2 and MaCCR3 are probably involved in lignin biosynthesis or lignification of banana. CAD gene family can be divided into three classes according to homology and affinity for substrates (Ferreira et al., 2019). In *Arabidopsis*, the functional characteristics of AtCAD1, AtCAD4, AtCAD5 and OsCAD2 have been clear, and they are involved in the process of lignin synthesis (Sibout et al., 2005; Zhang et al., 2006; Barakat et al., 2009; Hirano et al., 2012). In our results, MaCAD1 and MaCAD2 also have high homology, and are in the same subgroup as AtCAD4 with AtCAD5. MaCAD3, MaCAD4 and AtCAD1 belonged to the same class (**Figure 2F**), indicating that MaCAD1, MaCAD2, MaCAD3 and MaCAD4 play roles in lignin biosynthesis of banana.

As the main component of dietary fiber, lignin content in fruit has a great impact on fruit quality. During fruit ripening, a large number of degradation of cell wall substances lead to the destruction of cell wall structure and increase the cell gap and the soft in pulp (Chaplin et al., 1990). During fruit development and ripening, we found that 49 genes of lignin synthesis were expressed (RPKM>5), accounting for 47.11% (104 of the total), 24 genes were highly expressed (RPKM>50), accounting for 23.08%, and *MaC3H2* and *MaCCR-like7* were constitutively expressed (RPKM>30) at all the time points. We also found *Ma4CL-like9*, *MaHCT2*, *MaF5H4*, *MaC4H5* and *MaCCoAOMT2* were highly expressed (RPKM>100) at 14 DPH. Our results show that most of lignin biosynthesis genes are involved in lignin biosynthesis and the quality formation during banana fruit development and ripening stage.

Lignin is an important part of vascular plant cell wall and mainly deposits in the secondary wall cells of vascular tissue, mechanical tissue and protective tissue. It provides mechanical support for cells and tissues, facilitates the long-distance transportation of water and minerals, and forms a physical barrier with cellulose to resist biological and abiotic stresses (Lewis and Yamamoto, 1990). During growth, Cavendish banana is vulnerable to drought (van Asten et al., 2011), low temperature (Kang et al., 2007) and salt (Xu et al., 2014), resulting in the reduction of fruit yield and quality. In our results, we found that most lignin biosynthesis genes from *Musa acuminata* genome had low RPKM value. Only 24 genes were differentially expressed after low temperature, drought or salt stress treatments, among which, 14 genes were differentially expressed in only one of the above three stresses (**Figure 5**). Therefore, the inactive gene expression of lignin biosynthesis genes may be the cause of more vulnerable to abiotic stresses in Cavendish banana. PAL is the key enzyme and rate limiting enzyme of phenylpropane metabolism. The products of phenylpropane metabolic pathway play an important role in plant growth and development and response to stresses (Harakava, 2005; Huang et al., 2010). In banana fruit, *MaPAL* participates in heat pretreatment inducing chilling tolerance during postharvest (Chen et al., 2008). In our results, we found that MAPALs were involved in the response to low temperature, drought and salt stresses. *MaPAL1*, *MaPAL3*, *MaPAL4* and *MaPAL7* were up-regulated and differentially expressed in Cavendish banana responding to low temperature, drought and salt stresses, indicating that *MAPALs* are involved in Cavendish banana responding to abiotic stresses, and may have some redundancy in function, which is similar to the results of *AtPALs* in *Arabidopsis* (Huang et al., 2010).

Many studies have shown that lignin is involved in the construction of plant defense system, which is an important barrier for plants to prevent or reduce the invasion of pathogens (Boudet, 2000). In banana, treatment with elicitors from Foc R4 causes increased lignin deposition in roots of ‘Williams’ and ‘Goldfinger’ varieties, and higher contents of lignin are induced in the disease-resistant variety ‘Goldfinger’ (De Ascensao and Dubery, 2000). The lignin content accumulation of tolerance varieties is faster than that of susceptible varieties after inoculation with Foc TR4 (Van Den Berg et al., 2007). During Cavendish banana inoculated with Foc TR4, lignin biosynthesis genes such as *MAPALs*, *MaC4Hs*, *Ma4CLs* and *MaCADs* were significantly up-regulated and differentially expressed in cultivar Nongke No.1, which have acquired resistance to Foc TR4 through somaclonal variation from Cavendish banana (Wang et al., 2015). Up to now, GCTCV-119 is the best Foc TR4-tolerant alternative cultivar for the Cavendish (Ploetz, 2015). Pahang is highly resistant to Foc TR4 in the greenhouse and field trials, which is a wild germplasm that belongs to subspecies *Musa acuminata* ssp. *Malaccensis* (D'Hont et al., 2012; Zuo et al., 2018; Zhang et al., 2019). In our results, we found that 14 lignin biosynthesis genes were differentially expressed and significantly down-regulated in Cavendish banana variety, and only *MaF5H2* was significantly up-regulated, indicating that the expressions of lignin biosynthesis genes are not activated in susceptible variety responding to Foc TR4 infection. However, we found most lignin biosynthesis genes were significantly up-regulated and differentially expressed in both GCTCV-119 and Pahang varieties (**Figure 6**). Our results show that the expression of lignin biosynthesis genes is activated in banana resistance to Foc TR4 infection, which is consistent with the previous results (De Ascensao and Dubery, 2000; Van Den Berg et al., 2007).

WGCNA can identify functionally related or similar gene expression modules in high-throughput data, and we can consider gene function and its relationship from biological function as a whole, and we can specifically screen the co-expression modules with high biological significance with the target traits (Zhao et al., 2010). In our results, we found that 61.54% of lignin biosynthesis genes had co-expression network with 725 transcription factors from 68 gene families (**Figure 8**), suggesting that lignin biosynthesis genes may play important roles in the lignin biosynthesis for Cavendish banana during the growth or response to stresses. MYB, NAC (Nam, ataf1/2, CUC2) and LIM (LIN-11, ISL-1, MEC-3) transcription factors were related to the secondary wall synthesis (Zhao and Dixon, 2011). Overexpression of *MusaNAC68* can significantly reduce the expression of *MaPAL*, *Ma4CL*, *MaC4H*, *MaCOMT* and *MaCcOAMT*, resulting in reduced secondary wall thickness coupled with diminished lignin deposition (Negi et al., 2019). *MusaMYB31* as repressor of lignin biosynthesis in banana, overexpression of *MusaMYB31* in banana cultivar *Rasthali* (*Musa* cv. *Rasthali*) can reduce the deposition of lignin in Secondary wall (Tak et al., 2017). In the co-expression network, 97 *MaMYBs* and 34 *MaNACs* transcription factors were associated with lignin biosynthesis genes (**Figure 8** and **Supplementary Table 8**), showing that these transcription factors may be candidate genes for regulating lignin biosynthesis in banana. WRKY and ERF transcription factors are widely involved in the process of plant responding to stresses (Rehman and Mahmood, 2015; Chen et al., 2018). Based on the whole gene family analysis, several *MabHLHs* were involved in banana resistance to Foc TR4 infection (Wang et al., 2020). In our results, we also found large number of *MaERFs* (88), *MabHLHs* (70) and *MaWRKYs* (59) transcription factors were associated with lignin biosynthesis genes (**Figure 8** and **Supplementary Table 8**). P-coumarate 3-hydroxylase (C3H) determines the carbon source flow direction of lignin monomer and is also the rate limiting enzyme of phenylpropane pathway (Franke et al., 2002). Using yeast one hybrid and LUC activity assay, we also found that these transcription factors could bind with the promoter of *MaC3H2*, indicating that *MaMYB1*, *MaMYB2*, *MaMYB3*, *MaNAC1* and *MabHLH1* are active effectors of *MaC3H2* expression (**Figure 9**). In general, the above results suggest that these associated transcription factors in the co-expression network may play important roles in regulating lignin biosynthesis during fruit development, post-harvest ripening stages and response to stresses.

## Conclusions

We have developed a comprehensive resource of gene families involved in lignin biosynthesis in *Musa acuminata* genome. A total of 101 lignin biosynthesis genes were located on 11 different chromosomes. Most gene families had segmental duplication, and tandem replication was the main way to expand the number of *MaCOMTs*. We found most lignin biosynthesis genes were not involved in banana responding to abiotic stresses, except for *MaPALs*. Finally, the co-expression network of lignin biosynthesis genes was constructed using WGCNA to elucidate the abundant upstream regulatory networks of lignin biosynthesis in Cavendish banana. This comprehensive study improves our understanding of the lignin biosynthesis genes associated with fruit development, ripening processes, and stress responses and will establish a foundation for future studies on genetic improvement in banana.

## Data availability statement

All the data of RNA-seq have been uploaded to the CNSA (https://db.cngb.org/cnsa/) of CNGBdb with accession number CNP0000292. The accession numbers of all samples are listed in **Supplementary Table 2**.

## Author contributions

The study was conceived by ZW, B-yX and Z-qJ. Z-mW, X-mY, C-hJ, J-yW, and J-hL analyzed the lignin biosynthesis gene’s data from *Musa acuminata* genome and performed the bioinformatics analysis. ZW and C-hJ performed Foc TR4 inoculation, RNA isolation and qRT-PCR. ZW and J-yW wrote the manuscript. All authors contributed to the article and approved the submitted version.
